# Skeletal Accrual in Pediatric Growth Hormone Deficiency: A Narrative Review

**DOI:** 10.3390/cimb48100992

**Published:** 2026-09-25

**Authors:** Ignazio Cammisa, Eleonora Rulli, Margherita Zona, Chiara Veredice, Donato Rigante, Clelia Cipolla

**Affiliations:** 1 Department of Pediatrics, San Giovanni Evangelista Hospital, Tivoli, 00019 Rome, Italy; 2 Department of Life Sciences and Public Health, Fondazione Policlinico Universitario A. Gemelli IRCCS, 00168 Rome, Italy; 3 Pediatric Neurologic Unit, Fondazione Policlinico Universitario A. Gemelli IRCCS, 00168 Rome, Italy; 4 Università Cattolica Sacro Cuore, 00168 Rome, Italy

**Keywords:** growth hormone deficiency, children, bone health, fracture, skeletal integrity

## Abstract

Growth hormone deficiency (GHD) is a frequent cause of impaired linear growth and may also affect skeletal development and bone mass acquisition. This narrative review summarizes the current evidence on bone health in pediatric GHD, with a focus on clinical findings, response to recombinant human growth hormone (rhGH) therapy, and practical approaches to bone assessment. Available pediatric studies consistently show decreased BMD and bone mineral content at diagnosis, often involving the lumbar spine and total body, together with alterations in bone structure. During rhGH treatment, gradual improvements in bone turnover markers and BMD are generally observed, although responses are variable and may depend on age, growth response, and treatment intensity. Full normalization is achieved only in a subset of patients, highlighting heterogeneity in skeletal recovery. Given these findings, a structured clinical approach is recommended. An integrated algorithm for bone health evaluation in children with GHD should include biochemical assessment (IGF-1, calcium-phosphate metabolism, vitamin D status, and bone turnover markers) plus imaging evaluation, primarily using dual-energy X-ray absorptiometry (DXA) with appropriate pediatric and size-adjusted interpretation. Early initiation of rhGH therapy, alongside optimization of calcium and vitamin D intake, is essential to support bone accrual. Overall, pediatric GHD is associated with impaired skeletal development, but timely diagnosis and standardized monitoring through a combined clinical, biochemical, and imaging algorithm can improve the final bone outcomes. Further prospective studies are needed to refine assessment tools and optimize long-term management strategies.

## 1. Introduction

Growth hormone deficiency (GHD), characterized by inadequate secretion of growth hormone (GH) from the anterior pituitary gland and reduced peripheral production of insulin-like growth factor 1 (IGF-1), is one of the main causes of disrupted linear growth in children [1]. Longitudinal bone growth occurs through the process of endochondral ossification, in which the cartilaginous template is progressively replaced by mineralized bone. This process starts from the growth plate, where chondrocytes undergo sequential stages of differentiation across the resting, proliferative, and hypertrophic zones [2]. In this context, GH and IGF-1 act on multiple cell populations within the bone microenvironment, including mesenchymal stem cells (MSCs), osteoblasts, osteocytes, and osteoclasts, as summarized in Figure 1. GH and IGF-1 stimulate MSC proliferation and promote commitment toward the osteogenic lineage while inhibiting adipogenic differentiation, partly through activation of Wnt signaling and induction of osteogenic transcription factors [3,4,5,6,7,8]. In addition, the GH/IGF-1 axis plays a central role in osteoblast differentiation and function by activating signaling pathways involved in osteoblastogenesis, collagen synthesis, alkaline phosphatase activity, and osteocalcin secretion, essential for bone matrix formation and mineralization [3,4,5]. Furthermore, GH and IGF-1 contribute to radial bone growth and skeletal strength through osteocyte-mediated mechanisms [3]. These hormones also regulate osteoclast formation and activity, thereby participating in the coupling between bone formation and bone resorption, as reported by animal and clinical studies in which elevated GH/IGF-1 levels have been associated with increased bone turnover, whereas IGF-1 deficiency impairs osteoclast differentiation and resorptive capacity [3,4,5,6,7,8].

Therefore, considering the crucial role of the GH/IGF-1 axis in skeletal development, bone mineralization and remodeling, it is conceivable that GHD may negatively affect not only longitudinal growth but also overall bone health during childhood, a critical period for bone acquisition and attainment of peak bone mass and skeletal strength. However, the extent to which childhood GHD affects bone integrity and fracture risk remains incompletely understood, with available clinical evidence often showing heterogeneous results. In this context, the aim of this narrative review is to summarize the current evidence regarding bone health in pediatric patients with GHD, with a particular focus on fracture risk and skeletal fragility. Moreover, the effects of GH replacement therapy on bone acquisition and long-term skeletal outcomes will also be discussed.

## 2. Literature Search

We conducted a narrative review of the literature to provide an overview of skeletal health and bone outcomes in children with GHD, with particular emphasis on bone mass acquisition, skeletal structure, the effects of rhGH replacement, and the potential long-term consequences of childhood-onset GHD. Given the relevance of skeletal accrual during childhood and adolescence to lifelong bone health, evidence from adult populations with childhood-onset GHD was also considered when relevant to the long-term skeletal consequences of GHD and rhGH replacement. A literature search was performed in PubMed using combinations of terms related to GHD, rhGH treatment, bone health, bone mineral density, bone mineral content, bone mineralization, bone geometry, bone microarchitecture, bone turnover, skeletal development, peak bone mass, and fracture risk. Studies involving both pediatric and adult populations were included when they provided relevant information on skeletal outcomes, the effects of GHD, or the response to rhGH replacement. No restrictions based on publication year were applied, and studies of different designs were considered. Studies not published in English and studies that did not provide relevant information on GHD, skeletal outcomes, or the effects of rhGH replacement were excluded. Given the narrative nature of the review, study selection was non-systematic and based on expert synthesis of the available literature. No formal systematic review methodology, quantitative synthesis, formal assessment of study quality or risk of bias, or meta-analysis was performed. The available evidence was therefore synthesized descriptively, with emphasis on the relationship between GHD and skeletal development during childhood, the effects of rhGH replacement on bone accrual, and the potential long-term implications for skeletal health.

## 3. Growth Hormone Deficiency and Skeletal Integrity

### 3.1. Animal Studies

The GH/IGF-1 axis plays a main role in bone growth and skeletal integrity. At the growth plate, GH stimulates chondrocyte differentiation and enhances local IGF-1 production, promoting longitudinal bone growth via autocrine and paracrine mechanisms [1,9]. Consequently, GHD impairs both bone acquisition during growth and long-term skeletal strength, as reported by preclinical studies.

Experimental studies showed that GHD mice, compared with GH-sufficient controls, had reduced bone size and mineralization, with compromised trabecular microarchitecture and lower mechanical strength [10,11]. In GHD rat models, with reduced GH, IGF-1 and Insulin-like Growth Factor-Binding Protein 3 (IGFBP-3), Dual Energy X-ray Absorptiometry (DXA) documented decreased Bone Mineral Density (BMD), with disruption of cortical collagen organization, which showed fewer and thicker fibrils, indicating that bone material properties contribute to fragility beyond changes in mineral density [12]. Other animal models further showed that absence of IGF-1 or its receptor causes severe growth restriction, impaired chondrocyte maturation, and profound deficits in both cortical and trabecular bone [13,14,15,16]. In contrast, models selectively reducing circulating IGF-1, such as GHRH receptor knockout, GH receptor knockout, or liver-specific IGF-1-deficient (LID) mice, largely preserve trabecular bone while selectively affecting cortical bone, highlighting the distinct roles of systemic versus local IGF-1 [13,14,15,16]. The role of GH replacement is less studied, although in some studies it partially ameliorates bone defects, with trabecular architecture remaining suboptimal [10,11]. Collectively, these preclinical data support the biological plausibility that disruptions of the GH/IGF-1 axis impair skeletal integrity also in children, though most clinical evidence comes from adult populations.

### 3.2. Clinical Evidence in Children

In childhood and adolescence, periods of rapid skeletal accrual and peak bone mass attainment, GHD impairs longitudinal growth and may lead to suboptimal peak bone mass, a key determinant of lifelong bone health [17,18,19]. Accordingly, low bone mass in adults with childhood-onset (CO) GHD largely reflects inadequate bone accrual during growth, whereas in adult-onset GHD, reduced bone mass arises primarily from altered bone remodelling [17,18,19]. In recent years, fewer studies have examined pediatric bone outcomes in GHD, but available data indicate consistent deficits at diagnosis and partial recovery with recombinant human growth hormone (rhGH) therapy. The main findings of the available pediatric studies are reported in Table 1.

Reduced lumbar spine (LS) and femoral neck volumetric Bone Mineral Density (vBMD) in GHD children have been reported by Högler et al. and Van der Sluis et al., while Schweizer et al., focusing on bone geometry and cortical structure, observed reduced cortical area and thickness together with enlarged marrow area at diagnosis [20,21,22,23]. Moreover, Shore et al. demonstrated that children with GHD had lower-than-predicted Bone Mineral Content (BMC), reduced bone width, and lower BMC relative to body weight [24]. Delayed skeletal maturation accounted for approximately 35% of this deficit. These observations are in line with the findings of Baroncelli et al., who confirmed at diagnosis reduced lumbar spine BMC, areal BMD (BMDa), and several size-corrected density parameters, including BMD height, BMD-BMI, and volumetric BMDa. Their results suggested that bone volume was more severely impaired than bone area and that volumetric BMD correlated poorly with anthropometric variables, supporting the presence of a true deficit in bone mineralization [25,26,27].

Pediatric data point out that rhGH therapy could partially restore bone mass during growth. Högler et al. showed that after 24 months of rhGH treatment, markers of bone turnover and overall bone mass increased while total body BMC gain remained below expected levels, with modest improvements in vBMD [20]. Similarly, in the study of Van der Sluis et al., lumbar bone mineral apparent density (BMAD), once corrected for body size, normalized after the first year of treatment, followed later by normalization of LS-BMD and total body BMD values [21]. Comparable findings were reported by Boot et al., who documented significant increases in lumbar spine and total body BMD as well as BMAD during 2–3 years of rhGH therapy, with lumbar spine BMD SDS already showing improvement within the first six months of treatment [22]. In contrast, Schweizer et al. documented after 12 months of GH therapy that cortical density decreased, whereas the other structural parameters remained substantially unchanged [23]. Further evidence of the beneficial effects of GH replacement came from Kandemir et al. and Saggese et al., both of whom reported significant increases in lumbar spine BMD standard deviation score (SDS) after 6–12 months of treatment. Height gain appeared to contribute substantially to the observed improvement in bone mass, and nearly 46% of children achieved complete recovery of bone density parameters during follow-up [28,29]. Finally, Zamboni et al. highlighted the importance of treatment regimen, demonstrating a dose-dependent skeletal response to GH therapy. Daily GH administration produced greater improvements in BMC, the BMC-to-bone-width ratio, height velocity, and BMD compared with administration three times weekly, suggesting that continuous GH exposure may optimize bone accrual and skeletal development [30]. Reduced bone turnover, characterized by a greater decrease in bone formation than in resorption, is considered a major factor contributing to the lower aBMD observed in individuals with GHD. GH therapy stimulates bone remodeling units, expands the remodeling space, and enhances bone area, periosteal formation, and overall turnover [31,32,33]. This mechanistic framework may explain the delayed and time-dependent skeletal response observed in clinical studies.

### 3.3. Long-Term Outcome of GHD in Adults

#### 3.3.1. Consequences of Childhood-Onset GHD in Adulthood

The long-term consequences of GHD become particularly relevant in adulthood in individuals with childhood-onset (CO) GHD, particularly when GHD remains untreated or skeletal accrual during growth is substantially impaired. This reflects the importance of childhood and adolescence as critical periods for bone mass acquisition and attainment of peak bone mass [31,32]. Therefore, adult skeletal findings in individuals with CO-GHD provide relevant information on the long-term consequences of impaired skeletal development during childhood. Histological studies support the persistence of skeletal abnormalities beyond the growth period. Bravenboer et al., comparing transiliac bone biopsies from healthy adults and adults with CO-GHD, documented increased trabecular bone volume, a higher proportion of eroded surfaces, greater osteoid thickness, and prolonged mineralization lag time, findings consistent with delayed bone mineralization in CO-GHD [34]. Other studies have suggested that the lower BMD observed in adults with CO-GHD may partly reflect their shorter stature compared with age-matched controls. However, BMD remained significantly lower even after adjustment for height, suggesting that impaired bone accrual cannot be fully explained by differences in body size [35]. The effects of rhGH replacement in individuals with juvenile-onset GHD also suggest that skeletal deficits acquired during childhood may remain at least partially reversible. Several studies have reported increases in BMD following rhGH treatment in individuals with juvenile-onset GHD, potentially reflecting progressive mineralization of a previously immature skeleton, with changes observed as early as one year after treatment initiation [36,37,38]. These findings support the concept that restoration of GH action may contribute to continued skeletal maturation and mineralization beyond the period of linear growth.

#### 3.3.2. Adult-Onset GHD: Comparative Evidence

Adult-onset (AO) GHD provides a useful comparative context, as skeletal impairment in this setting occurs after peak bone mass has generally been achieved and is therefore characterized predominantly by alterations in bone remodeling rather than impaired bone acquisition during growth. In adults with GHD, short-term studies generally do not demonstrate significant increases in BMD following rhGH replacement, whereas more consistent improvements become evident after 12–24 months of treatment [39,40,41]. A meta-analysis by Davidson et al. documented progressive increases in lumbar spine BMD of 0.01 g/cm^2^ at 6–12 months, 0.02 g/cm^2^ at 18 months, and 0.03 g/cm^2^ at 24 months [42]. Long-term studies indicate that the greatest gains generally occur during the first 5–7 years of treatment, after which BMD tends to stabilize [40,41,43]. Different temporal patterns of BMD response have been described in adults with GHD. Some authors have proposed a triphasic response, characterized by an initial decline, followed by a gain after the first year and subsequent stabilization after approximately five years, whereas others have reported sustained increases for up to a decade of treatment [44,45]. The initial decline may reflect GH-induced activation of bone remodeling and expansion of the remodeling space, which can transiently attenuate BMC or BMD before measurable densitometric improvement occurs. Subsequent stabilization may partly reflect progressive dose adjustments that are insufficient to sustain further skeletal accrual [45]. The response to GH replacement in adults may also differ according to sex, with men generally showing greater increases in BMD than women. The mechanisms underlying this difference remain incompletely understood, although sex steroids may contribute. Physiological estrogen replacement in women with GHD has been reported to induce relative resistance to GH-stimulated IGF-I production, while estrogen may exert antagonistic effects on GH action at the peripheral tissue level [19,46,47,48].

Overall, the adult literature suggests that the skeletal consequences of GHD and the response to GH replacement differ according to the timing of GH deficiency. In CO-GHD, adult skeletal abnormalities may reflect incomplete bone acquisition during childhood and adolescence, whereas in AO-GHD, skeletal changes primarily reflect altered bone remodeling after peak bone mass has been attained. Across both settings, the skeletal response to GH replacement is generally slow, with remodeling-related changes preceding measurable increases in BMD.

## 4. Growth Hormone Deficiency and Fracture Risk

Reduced bone mass achievement and impaired skeletal development during childhood are considered the main determinants of osteopenia in adults with CO-GHD. Although untreated GHD is consistently associated with reduced bone mass, robust data on fracture incidence in children are insufficient [49]. No studies have specifically evaluated fracture risk in children with GHD, and extrapolation from adult cohorts is difficult. Importantly, there is currently no evidence demonstrating an increased fracture risk in children with isolated GHD. Only few studies have reported increased fracture incidence in adults with hypopituitarism and GHD, but their limited sample sizes preclude firm conclusions [50,51]. Notably, most adult studies included patients with not only GHD but multiple pituitary hormone deficiencies (MPHD), typically related to pituitary adenomas and surgery, and predominantly adult onset (AO) rather than CO-GHD. Therefore, these findings cannot be directly extrapolated to children with isolated GHD. Specifically, fracture rates have been reported to be approximately two- to threefold higher in patients with hypopituitarism and untreated AOGHD [52,53,54]. Although fracture risk appears greater in those with low areal BMD (aBMD), vertebral fractures have also been documented in patients with normal aBMD, indicating that aBMD alone may not reliably predict fracture risk [53,55]. Observational data suggest that GH replacement may reduce both vertebral and non-vertebral fracture risk. In large cohorts of hypopituitary adults, GH-treated patients exhibited lower incident fracture rates compared with untreated individuals, and a reduced fracture risk was observed in men—but not women—with AOGHD relative to population controls [55,56,57]. Overall, current evidence supports an increased fracture risk in AO MPHD, whereas data remain insufficient to demonstrate a clear excess risk in isolated CO or AO GHD [49,50,51]. In particular, the available evidence does not currently support an increased fracture susceptibility in children with isolated GHD, and the absence of pediatric fracture studies precludes definitive conclusions regarding long-term fracture risk. Individuals with long-standing severe isolated CO GHD or GH resistance do not appear to exhibit a markedly elevated fracture risk, suggesting that isolated GHD alone may not substantially increase fracture susceptibility [6].

Although the primary clinical use of rhGH is in patients with documented GHD, a few studies have explored its potential in osteoporosis unrelated to GHD, underscoring its role in bone. Randomized trials in postmenopausal women have shown that rhGH, particularly when combined with estrogen, calcium, and vitamin D, can increase bone mineral density and markers of bone formation, with some evidence of reduced fracture incidence over long-term follow-up from 56% to 28% [58,59]. However, these studies were generally small, heterogeneous, and produced modest or inconsistent effects on BMD, and the predictive value for fracture risk remains uncertain. Consequently, GH is not currently considered a standard therapy for osteoporosis in individuals without GHD, and further large-scale studies are needed to clarify its potential role in skeletal protection [60].

These findings underscore that GH exerts its most clinically relevant skeletal benefits in the context of deficiency, highlighting the importance of early diagnosis and appropriate replacement therapy to optimize bone mass acquisition, while emphasizing that evidence regarding its effect on actual fracture risk, particularly in children with isolated GHD, remains limited.

## 5. Potential Confounders of Skeletal Outcomes in GHD Children

Several biological, clinical, and treatment-related factors may influence skeletal outcomes in children with GHD and should therefore be considered when interpreting both the natural history of skeletal impairment and the response to rhGH therapy. Puberty is one of the main potential confounders, as it represents a critical period of bone mineral accrual and skeletal development [61]. Bone mass increases substantially during puberty through the combined effects of the GH/IGF-1 axis and sex steroids, with estrogen playing a particularly important role in the attainment of peak bone mass [62]. Consequently, differences in pubertal stage, timing of pubertal onset, and duration of sex-steroid exposure may substantially affect BMD and BMC independently of GH status or treatment. This is particularly relevant in children with GHD, in whom delayed puberty is relatively common and may prolong the period of reduced bone mineral accrual. Studies in patients with childhood-onset GHD have shown that untreated GHD during puberty may be associated with persistent reductions in adult bone density, whereas adequate GH and sex-steroid exposure during this critical period may contribute to more appropriate skeletal mineralization [31,63]. Moreover, sex-related differences in skeletal responses have been reported in patients with GHD, suggesting a complex interaction between GH and sex steroids. Estrogen directly influences bone remodeling and plays an important role in regulating the balance between bone formation and resorption during puberty. In males, the skeletal effects of testosterone are mediated, at least in part, through its aromatization to estrogen [64,65]. Nutritional and mineral factors represent another potential source of variability. Calcium and vitamin D are essential for normal skeletal mineralization, and inadequate intake or vitamin D insufficiency may contribute to impaired bone accrual independently of GHD [66]. Although reduced concentrations of biochemical markers of bone formation and alterations in vitamin D metabolism have been reported in children with GHD before treatment, more recent pediatric data suggest that vitamin D deficiency is not necessarily more prevalent in children with GHD than in healthy controls. However, vitamin D deficiency may be associated with a less favorable response to GH therapy, highlighting the importance of considering baseline nutritional status when interpreting treatment outcomes [67]. In addition, studies have suggested that calcium and vitamin D supplementation during GH treatment may enhance bone mass accrual in children with GHD, although these findings require confirmation in larger studies [68]. Therefore, differences in dietary calcium intake, vitamin D status, and overall nutritional status may contribute to the heterogeneity of skeletal outcomes observed across pediatric GHD cohorts. The presence of multiple pituitary hormone deficiencies is another important potential confounder. Children with MPHD may have concomitant deficiencies of thyroid, gonadal, or adrenal hormones, each of which may independently influence bone development and remodeling. In particular, untreated hypothyroidism and hypogonadism may adversely affect skeletal maturation and mineralization, whereas appropriate hormone replacement may modify the skeletal phenotype. Consequently, skeletal abnormalities observed in MPHD cannot necessarily be attributed exclusively to GH deficiency. Nevertheless, studies comparing isolated GHD and MPHD have reported broadly similar increases in bone turnover and BMD during GH therapy, although differences in pubertal development, sex-steroid replacement, age at treatment initiation, and duration of follow-up may contribute to the observed variability [28,69].

Treatment characteristics should also be considered when interpreting skeletal outcomes. GH dose, treatment duration, cumulative GH exposure, and timing of treatment initiation may influence the skeletal response, which evolves over time from an initial increase in bone turnover to progressive bone mineral accrual. Consequently, differences in treatment duration and GH dose may contribute to heterogeneity across studies. Long-term pediatric studies have demonstrated progressive improvements in BMD during GH therapy, with greater benefits associated with prolonged and adequate treatment exposure, whereas suboptimal treatment may be associated with lower bone mass at final height [29,70]. Treatment adherence represents another potentially modifiable determinant of GH exposure. Missed injections may reduce cumulative GH exposure and attenuate the expected benefits of therapy. Adherence may vary according to age, treatment duration, treatment burden, and patient- and family-related factors, with poorer adherence frequently reported during adolescence and long-term treatment [71,72,73]. Although most adherence studies have focused on growth rather than skeletal outcomes, suboptimal adherence may contribute to variability in the long-term skeletal response to GH therapy. Therefore, skeletal outcomes should be interpreted in the context of treatment dose, duration, and adherence, as well as growth and skeletal maturation.

## 6. Limitations and Future Directions

The available evidence on bone health in GHD is limited by the small sample sizes and substantial heterogeneity of the available studies. Study populations differ considerably in age, pubertal status, severity and duration of GHD, treatment duration and regimen, and the presence of additional pituitary hormone deficiencies, making direct comparisons across studies difficult. Moreover, several studies included adult patients with multiple pituitary hormone deficiencies, limiting the extent to which their findings can be extrapolated to children with isolated GHD. Another important limitation is the predominant reliance on DXA-derived areal BMD (aBMD) as the main measure of skeletal health. In children with GHD, particularly those with short stature or delayed skeletal maturation, aBMD may be influenced by bone size and therefore may not accurately reflect true volumetric bone density. More importantly, aBMD provides limited information on bone geometry, cortical and trabecular microarchitecture, and bone material properties. Only a limited number of pediatric studies have incorporated volumetric or structural assessments, and evidence regarding bone microarchitecture and bone quality remains scarce. Finally, clinically relevant fracture outcomes are insufficiently characterized in children with GHD. Long-term prospective pediatric studies evaluating peak bone mass attainment and fracture incidence into adulthood are lacking, and most available fracture data derive from adult cohorts with hypopituitarism, frequently including multiple pituitary hormone deficiencies and adult-onset GHD. Therefore, the extent to which the alterations in bone mass and structure observed in childhood translate into an increased lifetime fracture risk remains uncertain. Future research should focus on large, longitudinal studies in children with GHD assessing not only BMD but also bone microarchitecture, bone quality, and clinically relevant fracture outcomes. The integration of advanced imaging techniques and standardized, size-adjusted skeletal parameters may help to better characterize the long-term skeletal consequences of childhood GHD and clarify the effects of rhGH therapy during critical phases of skeletal development.

From a clinical perspective, an assessment of bone health may be considered in children with a new diagnosis of GHD, particularly in those with additional risk factors for impaired skeletal health. Depending on the clinical context, biochemical evaluation may include assessment of IGF-1, calcium-phosphate metabolism, vitamin D status, alkaline phosphatase, parathyroid hormone, and, where clinically appropriate, bone turnover markers such as Procollagen Type 1 N-Terminal Propeptide (P1NP), osteocalcin, and C-Terminal Telopeptide of Type I Collagen (CTX). These investigations should be considered as potentially useful tools rather than as mandatory components of a standardized monitoring protocol, as specific pediatric recommendations regarding their routine use in GHD are not uniformly established. Similarly, DXA may be considered when clinically indicated to provide information on bone mineral parameters in children with GHD. When performed, DXA findings should be interpreted with appropriate consideration of body size, height, skeletal maturation, and pubertal stage. The potential need for repeat DXA should be individualized according to the clinical context and the information expected to be gained from the examination, rather than considered as a routine requirement for all children with GHD. Early initiation of rhGH therapy remains an important component of the management of children with confirmed GHD and may contribute to improving bone mass acquisition and skeletal development over time. Adequate calcium and vitamin D intake should also be ensured according to individual nutritional status and clinical needs. The examinations and approaches summarized in Figure 2 should therefore be regarded as a possible framework for the assessment and monitoring of skeletal health in children with GHD. Their use should be adapted to the individual clinical context, considering age, pubertal status, severity and duration of GHD, associated pituitary deficiencies, nutritional factors, and other potential risk factors for impaired bone health.

## 7. Conclusions

The GH/IGF-1 axis plays a fundamental role not only in longitudinal growth but also in skeletal development, bone mineralization, and maintenance of bone strength. Experimental studies documented that childhood GHD is associated with impaired bone acquisition, altered bone geometry, and reduced BMD, potentially compromising optimal peak bone mass attainment. Although recombinant human GH therapy improves bone parameters, skeletal recovery appears gradual and may remain incomplete in some patients, particularly in cases of delayed diagnosis, leading to long-term skeletal vulnerability in adulthood. Bone health should therefore be considered an integral component of the diagnostic and therapeutic management of children with GHD, rather than a secondary consequence of impaired growth. Accordingly, pediatric endocrinologists should include bone health assessment, combining clinical, biochemical, and imaging parameters, as part of the initial evaluation of children with newly diagnosed GHD. Future research should focus on large prospective longitudinal studies in children assessing not only BMD but also bone microarchitecture, bone quality, and clinically relevant fracture outcomes, defining the long-term skeletal consequences of childhood GHD and optimizing strategies for GH replacement therapy during critical phases of skeletal development.

## Figures and Tables

**Figure 1:**
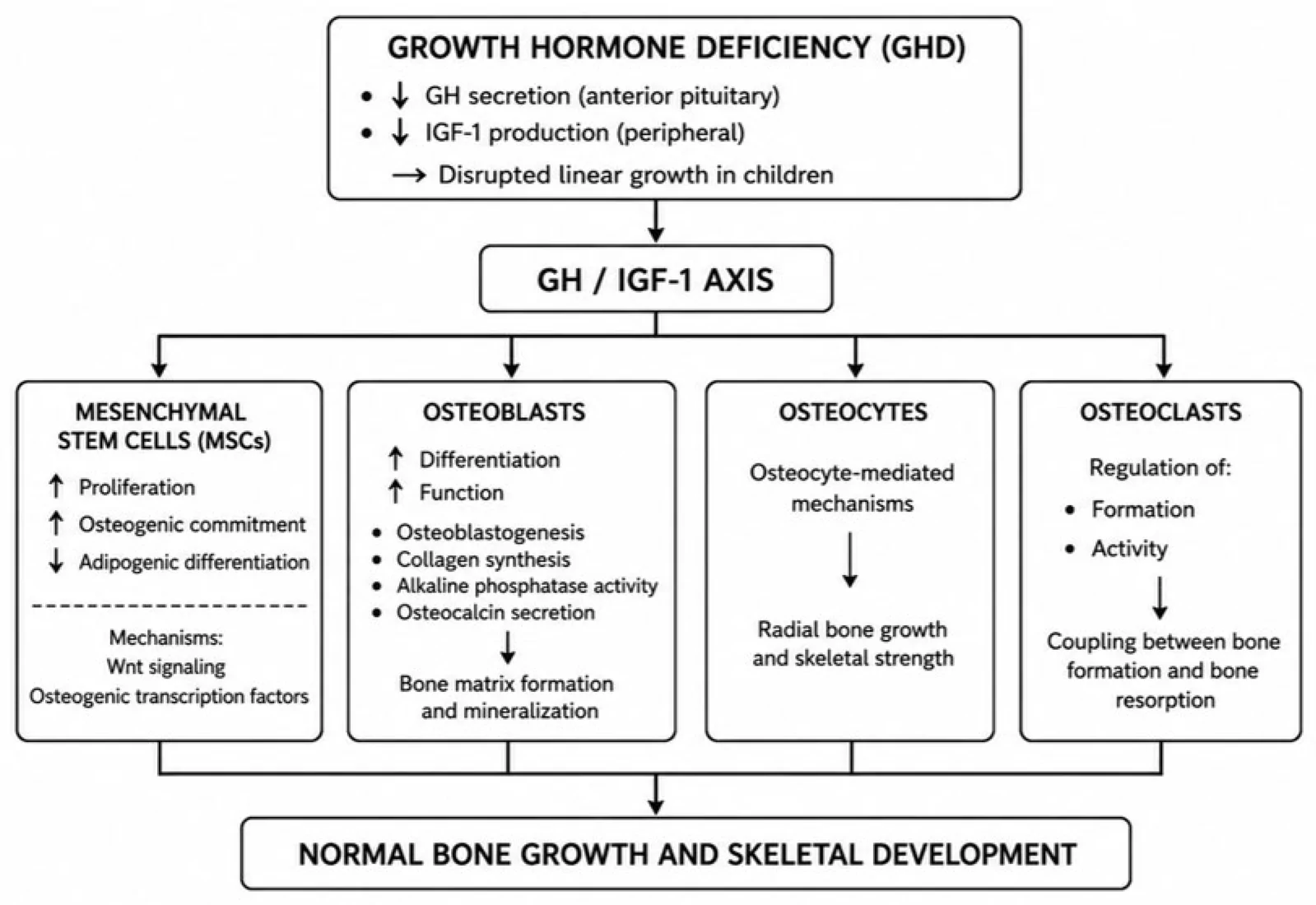
Overview of the role of the GH/IGF-1 axis in bone growth and skeletal development.

**Figure 2:**
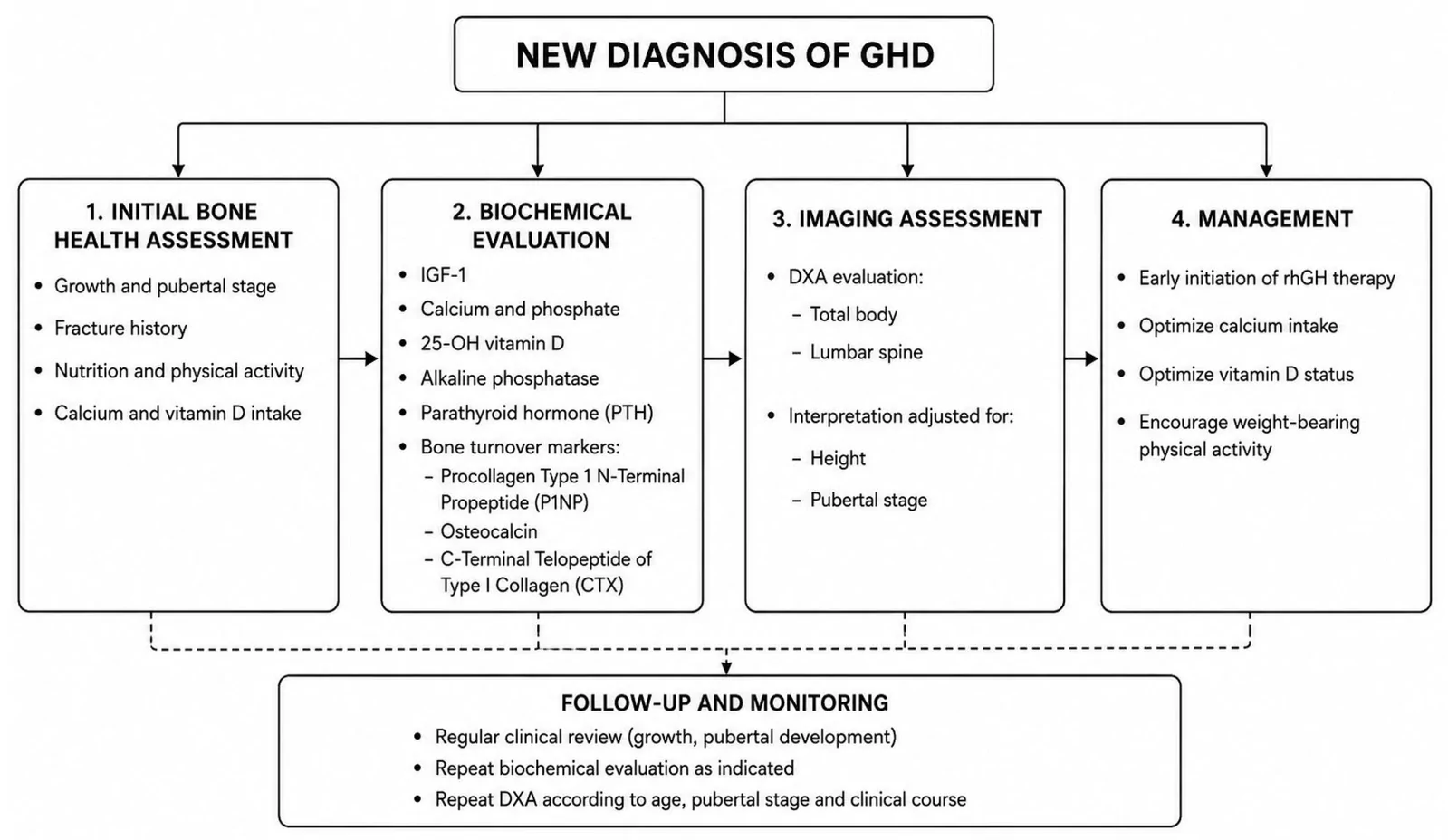
Possible framework for the assessment and monitoring of skeletal health in children with GHD.

**Table 1:** Clinical studies of bone health in children with growth hormone deficiency (GHD).

Study	Sample Size (N)	Mean Age (Years)	Follow-Up (Years)	Main Findings
Högler et al. [20]	20	9.42	2	At diagnosis: LS-vBMD SDS and FN-vBMD SDS were decreased, TB-BMC SDS was above average. During therapy: Increased bone turnover and mass, TB-BMC below expectations, minimal improvement in vBMD.
Van der Sluis et al. [21]	59	0.4–16.9	6	At diagnosis: LS-BMD, LS-BMAD and TB-BMD were reduced at baseline. During therapy: LS-BMAD increased to normal values after 1 year; LS-BMD and TB- BMD normalized 1 year later.
Boot et al. [22]	40	7.9	2–3	At diagnosis: LS-BMD, BMAD and TB-BMD were decreased. During therapy: LS-BMD, TB-BMD and BMAD increased; lumbar spine BMD SDS improved within 6 months.
Schweizer et al. [23]	45	7.5	1	At diagnosis: reduced cortical area/thickness and increased marrow area. During therapy: cortical density decreased, other measures stable.
Shore et al. [24]	18	NA	NA	At diagnosis: BMC, bone width, and BMC/body weight below predicted. During therapy: BMC, BW, and BMC/BW increased proportionally to height/weight gains.
Baroncelli et al. [25,26,27]	22	6.1–8.0	NA	At diagnosis: reduced LS-BMC, BMDarea, and size-corrected BMD.
Kandemir et al. [28]	39 (28 GHD and 11 MPHD)	10.4 ± 4.09	1	LS-BMD SDS significantly increased after 6 and 12 months of rhGH, with baseline height SDS as the main predictor of baseline BMD SDS. Improvement in height during therapy was positively associated with the increase in BMD.
Saggese et al. [29]	26	8.4 ± 2.4	1	At diagnosis: reduced bone density relative to chronological age, height, and bone age. During therapy: BMD significantly improved with complete recovery in 46.2% of the children.
Zamboni et al. [30]	22	5–8	Not specified	At diagnosis: BMC and BMC/BW were significantly lower. During therapy: bone parameters increased only in the group receiving a more intensive treatment regimen (daily regimens).

BMC (Bone Mineral Content), BMC/BW (Bone Mineral Content to Bone Width ratio), BMAD (Bone Mineral Apparent Density), BMD (Bone Mineral Density), BMDarea (Areal Bone Mineral Density), FN-vBMD SDS (Femoral Neck volumetric Bone Mineral Density Standard Deviation Score), LS (Lumbar Spine), rhGH (recombinant Human Growth Hormone), SDS (Standard Deviation Score), TB (Total Body), vBMD (volumetric Bone Mineral Density).

## Data Availability

No new data were created or analyzed in this study. Data sharing is not applicable to this article.
